# Short-Term Adverse Effects of the Fourth Dose of Vaccination against COVID-19 in Adults over 40 Years of Age

**DOI:** 10.3390/vaccines12040400

**Published:** 2024-04-10

**Authors:** Jussara Malo-Castillo, Harold Jiménez-Álvarez, Victor Ludeña-Meléndez, Solange Sarasvati Mayor Castro, Sheyla Rodríguez, Paula Ishikawa-Arias, Cristhian Terrones, Leonardo Ledesma Chavarría, Edgardo Linares Reyes, Virgilio E. Failoc-Rojas

**Affiliations:** 1 Sociedad Científica de Estudiantes de Medicina de la Universidad Nacional de Trujillo, Universidad Nacional de Trujillo, Trujilo 13011, Peru; jmaloc@unitru.edu.pe (J.M.-C.); t011801420@unitru.edu.pe (H.J.-Á.); t011801720@unitru.edu.pe (V.L.-M.); t051802220@unitru.edu.pe (S.S.M.C.); t511802620@unitru.edu.pe (P.I.-A.); 2 Facultad de Medicina, Universidad Nacional de Trujillo, Trujillo 13011, Peru; t511802020@unitru.edu.pe (S.R.); t051801220@unitru.edu.pe (C.T.); lledesma@unitru.edu.pe (L.L.C.); elinaresr@unitru.edu.pe (E.L.R.); 3 Facultad de Medicina, Universidad Cesar Vallejo, Piura 20001, Peru

**Keywords:** COVID-19, vaccines, adverse effects, Peru

## Abstract

Booster vaccines are a strategy to mitigate the conditions in the health, social, and economic fields that the COVID-19 pandemic has brought. A series of adverse effects have been observed since the first vaccination. The present investigation aims to describe the short-term adverse effects of the fourth dose against COVID-19 in adults older than 40 from a region of Peru. The study population was over 40 years of age at the COVID-19 vaccination center in Trujillo, Peru. A 21-day follow-up was conducted from vaccination with the fourth dose, considering sex, age, body mass index, comorbidities, history of COVID-19 infection, vaccination schedule, and simultaneous vaccination against influenza as variables of interest. Multinomial logistic regression with robust variance was used to estimate the risk ratio (RR). In total, 411 people were recruited, and it was found that 86.9% of the participants presented adverse effects after injection with the fourth dose of the vaccine against COVID-19. Pain at the injection site was the most reported symptom after 3 days. Assessment of adverse effects after 3 days found that age ≥ 60 years was associated with a lower likelihood of adverse effects compared to those younger than 60 years (RRc: 0.32; 95% CI: 0.0.18–0.59), males compared to females were associated with a lower likelihood of adverse effects (RRc: 0.54; 95% CI 0.30–0.98), being overweight (RRc: 2.34; 95% CI: 1.12–4.89), and last vaccine with Pfizer-BioN-Tech (RRc: 0.42; 95% CI: 0.18–0.96). Associated adverse effects are mild to moderate. Injection site pain and general malaise are the most frequent adverse effects.

## 1. Introduction

The coronavirus disease (COVID-19) pandemic has generated countless human losses and has affected almost all countries, not only in the health field but also in the social and economic field. Until mid-May 2023, worldwide, there were more than 680 million confirmed cases and about 7 million deaths caused by this disease, while in Peru, more than 4.5 million people were infected and a further 220 thousand died [1,2]. Comparing the quarters with the lowest and highest excess mortality rates reveals that, in Peru, the incidence of COVID-19 was associated with per capita income and the unemployment rate as the main risk factors. In contrast, the main protective factors were per capita health spending, access to health care, and health insurance in high-income countries [3].

SARS-CoV-2 infection is a respiratory disease and the difficulty in its containment lies in the capacity for mutation, especially at the level of the spike (S) protein in its N-terminal (NTD) and receptor-binding (RBD) domains, which has allowed us to recognize a series of variants of this virus, which can increase the rate of infection, virulence, and viral resistance [4]. 

Currently, vaccines are essential to prevent increased morbidity and mortality from COVID-19, due to their ability to reduce the elements that could cause infection in addition to avoiding a serious clinical picture. The effectiveness of the BNT162b2 vaccine with the fourth dose, compared to the third dose, is to be greater than 78% in preventing mortality after COVID-19 [5]. However, from the first inoculation to the present day, several short-term side effects have been observed. 

A randomized clinical trial found that local pain and fatigue were the most common adverse effects after a fourth dose of BNT162b2 or mRNA-1273 [6]. Govorkova et al. described that the most common adverse effects include mild symptoms such as pain, swelling, and redness in the area where the injection was applied, in addition to systemic reactions after the vaccine such as headache, drowsiness, or fatigue [7]; in another study, it was observed that 30% of those surveyed presented at least one adverse effect, 24.8% reported local reactions to vaccination, and 16.6% reported systemic reactions, with the most common adverse effects being local pain, fatigue, and general malaise [8]. In addition, isolated cases of severe symptoms have been reported as cases of severe acute myocarditis after receiving the third dose of the vaccine [9]. 

According to the data published by the Ministry of Health Peru (MINSA), it was observed that 30% of those surveyed presented at least one adverse effect, 24.8% reported local reactions to vaccination, and 16.6% reported systemic reactions, with the most common adverse effect being local pain, fatigue, and general malaise [8]. According to data published by the MINSA, until February 2023, it was reported that pain in the injection area, headache, pyrexia, and malaise are the main adverse effects attributed to vaccination, and of these, 81.8% of the reported cases were mild, 17.7% moderate, and only 0.5% of a serious nature. These data highlight the main adverse effects secondary to vaccination according to the Pharmacovigilance Report: supposedly at-tributed adverse events to vaccination or immunization (ESAVI) reported against vaccines BBIBP-CorV (Sinopharm), Comirnaty–Pfizer, Vaxzevria–AstraZeneca and Spikevax–Moderna; however, they failed to have a serious impact [10]. Vaccination has continued on a large scale throughout the country, to date, with the option of applying for the fourth dose. The inoculation started on 9 February 2021, with the health personnel being the first to receive it. Subsequently, the vaccination was opened up to the public; the first to receive it were adults over 70 years of age who had waited five months after receiving the third dose [11]. 

Although many studies have been conducted in different parts of the world, to our knowledge, few studies have addressed the adverse effects of the fourth dose in Peru. For example, the studies carried out by Bautista and Chipana [12], Gironzini Cordova [13], and Malca Díaz [14] only covered the study of the adverse effects of the first dose, the first two doses, or up to the third dose. The reasons for having approached the study lie in different cultural and geographical contexts, identifying possible variations and contributing to the scientific literature. In addition, the results can help improve dissemination from health services to communities that are hesitant to get vaccinated for fear of having serious adverse effects. An adequate investigation requires follow-up and monitoring to obtain figures that support the safety of the fourth dose, in terms of the severity or not of the adverse effects of vaccination, in our context, which is why the present investigation aims to describe the main short-term adverse effects of the fourth dose against COVID-19 in adults over 40 years of age from a region of Peru to assess the risks associated with the adverse reactions of the fourth dose against SARS-CoV-2.

## 2. Materials and Methods

### 2.1. Study Design

A prospective observational cohort study was conducted, with a target population consisting of adults aged 40 years or older who attended between 4 June and 1 July 2022 to receive the fourth dose of mRNA, BNT162b2 (Pfizer, NY, USA) or ARNm-1273 (Moderna, MA, USA), at one of the 8 points of vaccination of the city of Trujillo: Health Center (CS) Unión, CS Sagrado Corazón, CS Aranjuez, CS Jardines, CS San Martín, CEPUNT, Parque Vallejo and Plaza de Armas. Trujillo is the capital of the La Libertad region, located in northern Peru. Trujillo has a population of 1,005,395 inhabitants, which represents 2.9% of the entire Peruvian population; the population over 40 years of age in Trujillo is 337,474 (33.6% of the entire population of Trujillo) (source: https://www.datosabiertos.gob.pe/dataset/poblaci%C3%B3n-peru (accessed on: 15 November 2023)). In Peru, until September 5, 2023, 21,385,427 third-dose vaccines have been given to people over 12 years of age (74.8% of the target population), with only 752,515 doses in Trujillo, which represents coverage with the third dose of 82.14% in that place (source: https://www.minsa.gob.pe/reunis/data/vacunas-covid19.asp (accessed on: 15 November 2023)).

### 2.2. Procedure

The participants were directly recruited at the vaccination centers by taking a sample of consecutive cases. Participants who did not comply with the four doses of COVID-19 and those with a history of organ transplantation, and severe clinical conditions (defined as any person with diagnosed cancer, HIV, hepatitis B, or immunosuppressive therapy treatment) were excluded. The volunteers signed a document that accredits their informed consent to be included in the study, in the follow-up, and in the evaluations to be studied. They also filled out the contact form that included variables of interest such as their age, sex, occupation, educational level, vaccination schedule against COVID-19, presence and type of comorbidities, history of previous COVID-19 infection, and whether they had been vaccinated Simultaneously against influenza. Comorbidity was defined as the presence of one or more of these diseases confirmed by medical diagnosis of: AHT, DM2, rheumatic diseases (rheumatoid arthritis, osteoarthritis, fibriomyalgia), respiratory diseases (asthma, COPD), cardiovascular diseases, neurology diseases, and chronic kidney diseases. Subsequently, participants were monitored on days 3, 7, 14, and 21 after the day of inoculation with the fourth dose using a Google Form survey (Appendix A) sent by WhatsApp^®^ or by phone call as previously agreed with the participants. The data collected were compiled in Microsoft Excel^®^ where a code was assigned to each participant to protect her identity. The survey included topics such as the presence of adverse effects (AEs) at the level of the arm where they received the vaccine, such as injection site pain, injection site swelling, or armpit swelling near the injection site; and systemic AEs such as fatigue, headache, muscle pain, fever above 38 °C, fever below 38 °C, nausea or vomiting, diarrhea, general malaise, widespread rash, rash on the face, chest pain, irregular pulse, and shortness of breath. All of these response options were chosen because they were previously reported in vaccine clinical trials. Due to the design of the study, the investigators who collected the data were different from those who carried out the follow-up to avoid information bias.

Participants had the option of providing free text responses to detail any unspecified symptoms. If there were any adverse effects, the question of duration was also included. To compare health status after the third and fourth doses, questions on the presence of effects after the third dose against COVID-19 and their perception of both statuses in comparison were included. Data collected between 7 June and 22 July were included for statistical analysis.

The sample was calculated in EPIDAT 4.2 (*Servicio de Epidemiología de la Dirección Xeral de Saúde Pública da Consellería de Sanidade*, Galicia, Spain) at 95% confidence, considering the data from the Regional Government of La Libertad on the number of inhabitants over 40 years of age in the city of Trujillo able to receive the fourth dose against COVID-19 (328,119 inhabitants), with an expected rate of 35% according to background information, a precision of 5%, and a maximum of 20% in type II error. The minimum sample was 351.

### 2.3. Statistical Analysis

In the descriptive analysis, frequencies and percentages were used for the categorical variables and measures of central tendency and dispersion (mean and standard deviation or median and interquartile range) according to the evaluation of normality. In the bivariate analysis, the Chi-square test or Fisher’s exact test was used to assess differences between categorical variables in adverse events (yes/no) in the study period. We set a significance level of 5%.

To find the association between the risk variables of interest and the persistence of adverse reactions according to observation time, multinomial regression models were constructed according to the presence of symptoms at 3, 7, and 14 days. Crude and adjusted multinomial logistic regression were performed for all models, and association measures with their respective 95% confidence intervals were reported; in the Stata statistical package, we requested the relative risk report using the *rrr* command. The variables included in the adjustment were age, sex, homologous or heterologous vaccination, presence of comorbidities, history of previous COVID-19 infection, and simultaneous vaccination against influenza according to epidemiological criteria theoretical background found in the literature [15,16]. Multicollinearity was evaluated using variance inflation factors considering a cut-off point less than 0.05. The variables of last vaccination or previous BBIBP-CorV vaccination could not be added to the adjusted model because they had a high collision and collinearity with vaccination schedule and age.

For a sensitivity analysis, Cox proportional hazards regression models were constructed to estimate hazard ratios (HRs) according to the duration time of adverse events and the different possible risk factors, and the 95% CI was also constructed, after evaluation of hazard ratio assumptions. The maximum observation time was defined as 21 days. These results are presented as Supplementary Table S1.

The statistical software program STATA v.17 (Stata Corporation, College Station, TX, USA) was utilized for data analysis.

### 2.4. Ethical Aspects

The study was reviewed and approved by the ethics committee of the National University of Trujillo with the code 224-2022-UNT-FM-CE. The necessary permits were obtained to carry out the project in health centers associated with the Ministry of Health in Peru. Participation was voluntary, independent of the health care facility, and participants could withdraw at any time from the study.

## 3. Results

Among the 411 included participants, 206 (50.1%) were men and 205 (49.9%) were women. The mean age was 57 ± 12 years, and the mean BMI was 28.1 ± 4.3. In total, 17.3% had at least one comorbidity (AHT, DM2, rheumatic, respiratory, cardiovascular, or other). Only seven patients (1.7%) required medical care (they attended for pain (three), and fever (four) and reported that they had taken acetaminophen). A history of COVID-19 infection was reported in 128 (31.1%) participants. All patients received their last dose a messenger RNA vaccine (Modern and Pfizer), the homologous vaccination scheme was characteristic of 281 (68.4%) respondents, and only 69 (16.8%) were vaccinated against influenza simultaneously (Table 1).

**Figure 1:**
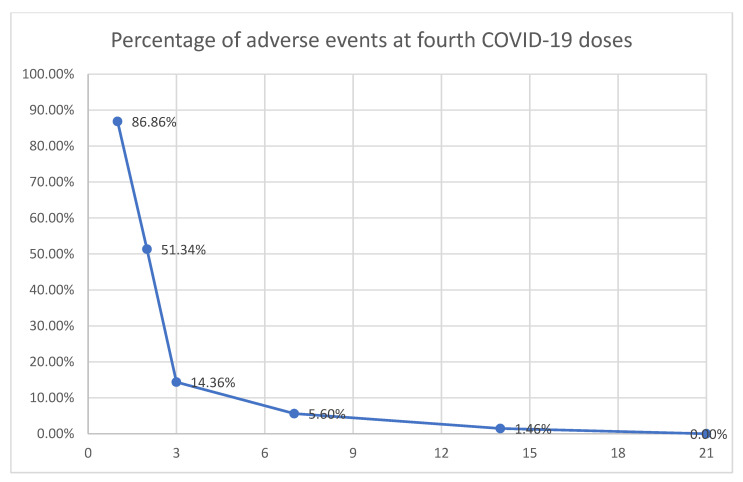
The frequency of adverse events from fourth COVID-19 doses over time in Trujillo, Peru.

On the first day, a considerable number of patients experienced adverse events, accounting for 86.9% (357 out of 411). However, after three days, there was a significant decline, with only 14.4%; the details of the percentages can be seen in Figure 1

In the present study, it was found that 86.9% of the participants presented adverse effects 3 days after inoculation with the fourth dose of the vaccine against COVID-19. More than half of the participants (52.6%) reported AEs both at the level of the arm where they were inoculated with the vaccine and at the systemic level. In total, 61.1% of those surveyed presented only one AE at the level of the arm, the most common being pain in the injection area (78.6%). On the other hand, among the 59.4% of participants who presented adverse effects at a systemic level, general malaise (33.3%), muscle pain (27.5%), and headache (20.5%) stood out. Regarding the duration of the symptoms, a period of two days (43.1%) predominates, followed by a duration of one day (40.9%) (Table 2). From Table 2, we can deduce that there was variation in the presence of adverse events, number of adverse events, and symptoms of adverse events over time, mainly decreasing when we evaluated it from day 3 to day 14.

In the bivariate analysis, age (*p* < 0.001), vaccination scheme (*p* = 0.026), last vaccine (*p* = 0.009), and sex (*p* = 0.043) stood out as the factors associated with the presence of adverse effects (Table 3).

The simple multinomial logistic regression model examines the association of multiple factors entering the model simultaneously (age, sex, BMI, presence of comorbidities, simultaneous influenza vaccination, history of previous COVID-19 infection, last vaccine, and homologous/heterologous). Assessment of adverse effects at 3 days found that age ≥ 60 years was associated with lower risk of adverse effects compared to those younger than 60 years (RRc: 0.32; 95% CI: 0.18–0.59), males compared to females were associated with lower likelihood of adverse effects (RRc: 0.54; 95% CI: 0.30–0.98), being overweight (RRc: 2.34; 95% CI: 1.12–4.89), and last vaccine with Pfizer-BioN-Tech (RRc: 0.42; 95% CI: 0.18–0.96). A similar observation was seen at 7 days, with the risk factors being age less than 60 years, heterologous vaccination, and vaccination with Moderna. For adverse events persisting up to 14 days, the risk factors were heterologous vaccination (RRc: 3.23; 95% CI: 1.14–9.11) and being overweight (RRc: 4.02; 95% CI: 1.28–12.65) (Table 4). 

The analysis also observed that patients who previously received the attenuated virus SARS-CoV-2 vaccine BBIBP-CorV (Sinopharm) in their vaccination schedule were at higher risk of having persistence of adverse events at 3 days (RR: 2.01; 95% CI: 0.94–4.64) and 7 days (RR: 2.88; 95% CI: 1.13–7.31).

History of COVID-19, simultaneous vaccination with influenza vaccine, and the presence of comorbidities did not seem to be associated with the presence of adverse effects from the fourth dose against COVID-19. According to the multiple multivariate regression model, at the first 3 and 7 days, age, sex, and vaccination schedule were a factor associated with the presence of adverse events to the vaccine; persistence up to 14 days was related to the heterologous vaccination schedule. The multiple logistic regression model for the presence of effects at the level of the arm where the fourth dose was inoculated with COVID-19 did not show significant associations.

In addition, a sensitivity analysis was performed to evaluate the association of possible risk factors in a Cox regression model. In the hazard ratio analysis, we found that age older than 60 years (HR: 0.77; 95% CI: 0.67–0.89) and male sex (HR: 0.88; 95% CI: 0.77–1.00) were associated with a lower hazard of adverse events. Concurrent vaccination with influenza (HR: 1.20; 95% CI: 1.04–1.40) and prior BBIBP-CorV (HR: 1.14; 95% CI: 1.01–1.30) were associated with an increased hazard of adverse events (Supplementary Table S1).

## 4. Discussion

Although the present study found a high incidence of adverse effects (AEs) after inoculation of the fourth dose against COVID-19, 84% of them lasted a maximum of 2 days, and only 1.7% of the participants requested medical attention.

The findings are like those of the safety and efficacy study by Polack et al. where most of the AEs were mild to moderate in severity and resolved in 1 to 2 days [15]. Compared with receiving the third COVID-19 dose, 74.7% of participants reported that their symptoms were similar or milder after receiving the fourth booster dose. Duy Chong et al. support that the AEs from booster vaccination are lower, and their incidence is the same as for the first or second vaccination [16]. These data seek to reduce the misinformation and hesitation that may arise in citizens not wanting to get vaccinated for fear of the severity and duration of the adverse effects [17].

The inverse association between the presentation of AD and the age of the patients is mainly justified by the deterioration of the immune response. Various studies have described how aging promotes immune system dysfunction due to the atrophy of lymphoid tissues responsible for generating virgin T and B lymphocytes involved in immunization with vaccines and, therefore, a decrease in immune reactions and hypersensitivity [17]. At the end of 2020, Baden et al. in a randomized trial evaluating the mRNA-1273 (Moderna) vaccine, supported that both injection-site and systemic AEs were more common among participants 18 to 65 years of age than those older than 65 years [18]. Likewise, in the pharmacovigilance study on the population of Peru, carried out by Vargas Espino [19], it was identified that events supposedly attributed to vaccines or immunization (ESAVI) against COVID-19 were more frequently reported in people between 30 and 60 years old, predominantly in females. However, it is important to highlight those studies carried out in a younger population, such as those carried out by Rodríguez Angeles [20] in children between 8 and 9 years old residing in a city in northeastern Peru, also report a high frequency of mild AEs.

On the other hand, simultaneous vaccination against influenza and presenting comorbidities were associated with the appearance of AEs. A cohort of self-reported data published in 2021 found that the simultaneous administration of a COVID-19 mRNA booster and an influenza vaccine was associated with 8% to 11% increases, respectively, in systemic response compared with a COVID-19 mRNA booster and COVID-19 alone [21]. It is relevant to take this variable into account due to the promotion of simultaneous vaccination against influenza because it is associated with a reduction in the risk of infection and severity of COVID-19 [22], and in addition, its co-administration is safe and produces immunogenicity; although, the data are still limited [23]. Regarding comorbidities, the findings are consistent with what was described by Majumder et al. where it was reported that diabetes, hypertension, and asthma were associated with a significantly higher risk of presenting AD, compared to having no comorbidities [24]. However, other investigations contrast this fact by stating that patients with diabetes indicate a diminished humoral immune response compared to healthy individuals [25], so a lower reactogenicity would be expected.

Injection site pain was the most common local AE, reported in 323 (78.6%) participants, and persisted in 15 (3.7%) of those surveyed up to day 14. The presence of local AEs in the injection site has previously been reported by Moya et al. [26]. However, these authors specifically associate improper vaccination technique with local adverse reactions that are persistent, not with all local AEs. This could affect the subacromial-subdeltoid bursa, the periosteum, or other neural structures, causing local adverse reactions of longer duration. Bernal et al. [17], reported persistent injection site pain for up to 21 days as part of a hypersensitivity reaction secondary to vaccination, which would occur more frequently and inversely with age, due to greater immunocompetence in younger patients [27].

General malaise (33.3%), muscle pain (27.5%), and headache (20.5%) were the most prominent symptoms at a systemic level, which coincides with the studies consulted [6,8] and the latest report by the Agency Spanish Medicines and Health Products [28]. It is urged to position reactogenicity in response to immunization as a “good sign” of immunocompetence, therefore, at a younger age, there is a greater probability of presenting more marked adverse effects [29].

A significant association was found between the development of systemic AEs and a history of COVID-19 infection (*p* < 0.02). This has been previously reported by Álvarez C. et al. [30] in their study carried out on health workers, where 50.7% and 30% of those who presented side effects after the first and second dose, respectively, had been previously diagnosed with COVID-19. However, even though the etiology of this apparent clinical association has not been fully elucidated, various authors such as Villar-Álvarez et al. [31] state that the benefits of vaccination outweigh the risks in patients with previous SARS-CoV-2 infection.

The females reported a higher frequency of nausea, headache, and general malaise. The hormonal interaction of estradiol may trigger greater immunogenicity and reactogenicity after vaccination [32,33]. This premise suggests that AEs are more recurrent in women than in men; however, sex was not significantly associated with the higher number of AEs. Additionally, fever less than 38 °C manifested more significantly in men.

An important limitation regarding the association between presenting symptoms and the fourth dose of COVID-19 in some patients is the addition of the influenza vaccination intervening variable since for ethical reasons participants were not prevented from having the influenza vaccination. If they did, some symptoms may be the result of either the influenza vaccine or a combination of both because the reported adverse effects of both the COVID-19 and influenza vaccines are similar [21].

Another possible limitation may be the nocebo effect, although the nocebo effect may have occurred, it is likely to have been minimized because the study participants voluntarily went to their vaccination center for vaccination, which may have created a state of complacency, and the vaccinating staff adequately explained the procedures, possible adverse effects, and what to do in case they were felt. A control group could not be obtained due to a lack of budget, time, and access to enough participants. However, our selection criteria and rigorous analysis will weigh the internal validity and conclusions of the study.

Additionally, since our study only included participants over 40 years of age, there is a higher rate of underlying medical problems and probably lower reactogenicity at the fourth dose of the vaccine; therefore, our results cannot be generalized to all age groups but rather limited to populations within the age range studied. However, our results may contribute to improving the booster vaccination rate, as concerns about adverse effects have been shown to generate significant hesitation. This hesitation can negatively impact not only the individual but also the general population, as it may contribute to the spread of the virus and an overload of healthcare services. Therefore, Peruvian health authorities are urged to disseminate information emphasizing issues such as the fear of adverse effects and the risk–benefit relationship that must be considered when getting vaccinated.

## 5. Conclusions

In conclusion, this study shows the frequency of symptoms attributed to the vaccine both at the level of the injection area and at the systemic level, the main ones being pain in the vaccination area, general malaise, muscle pain, and headache. However, it is important to report the presence of some moderate to high-risk symptoms, which were reported by patients but cannot be effectively attributed to vaccination due to insufficient evidence.

Although the persistence of symptoms was maintained only until the second week (14 days) post-vaccination, these symptoms were mild and did not represent a considerable risk to the health of patients older than 40 years, even when they presented comorbidities. In this way, our findings reaffirm the mildness of the adverse effects and the relative safety of the fourth booster dose with COVID-19, regardless of the type of vaccine.

## Figures and Tables

**Table 1:** Characteristics of adults over 40 years of age who came to be vaccinated with the fourth dose against COVID-19 in Trujillo, Peru (N = 411).

Characteristics	N°	%
**Sex**		
Female	205	49.9
Male	206	50.1
**Age (years)**		
Mean ± SD	57.2 ± 11.6	
40–49	127	30.9
50–59	120	29.2
60–69	96	23.3
≥70	68	16.6
**Level of study**		
Initial/Elementary	25	6.1
Secondary	104	25.3
Technique	41	10.0
University	241	58.6
**Occupation**		
Housewife/None	81	19.7
Retired	56	13.6
Dependent	220	53.5
Independent	54	13.2
**BMI**		
Mean ± SD	28.1 ± 4.3	
normal weight	99	24.1
Overweight	195	47.4
Obesity	117	28.5
**Number of comorbidities**		
0	340	82.7
1	61	14.9
2 or more	10	2.4
AHT	3.4	8.27
DM2	23	5.6
rheumatic	6	1.5
respiratory	3	0.7
cardiovascular	4	1.0
other	11	2.7
**History of COVID-19 infection**		
No	283	68.9
Yes	128	31.1
**Last vaccine**		
mRNA-1273 (Moderna)	379	92.2
BNT162b2 (Pfizer-BioNtech)	32	7.8
**vaccination scheme**		
Homologous	281	68.4
Heterologous	130	31.2
**Required medical care**		
No	7	1.7
Yes	404	98.3
**Simultaneous vaccination against influenza**		
No	342	83.2
Yes	69	16.8
**Previous BBIBP-CorV**		
No	305	74.2
Yes	106	25.8

SD: standard deviation; BMI: body mass index; AHT: arterial hypertension; DM2: type 2 diabetes.

**Table 2:** Description of the symptoms presented during the first 14 days of follow-up to adults over 40 years of age who came to be vaccinated with the fourth dose against COVID-19 in Trujillo, Peru (N = 411).

	Day 3		Day 7		*p*-Value *	Day 14		*p*-Value ±
	N°	%	N°	%		N°	%	
**They presented adverse effects**					0.025			<0.001
Only on the arm	113	27.5	35	8.5		14	3.4	
Systemic level only	28	6.8	26	6.3		10	2.4	
Both	216	52.6	20	4.9		2	0.5	
None	54	13.1	330	80.3		385	93.7	
**Number of adverse effects in the arm**					0.321			<0.001
0	82	20	356	86.6		395	96.1	
1	251	61.1	50	12.2		16	3.9	
2 or more	78	18.9	5	1.2		0	0	
**Adverse effects on the arm**					<0.001			<0.001
Injection site pain	323	78.6	49	11.9		15	3.7	
Swelling at the injection site	71	17.3	3	0.8		0	0	
Swelling in the armpit, close to the injection area	9	2.2	1	0.3		0	0	
Another reaction	11	2.7	7	1.7		1	0.2	
**Number of systemic adverse effects**					<0.001			<0.001
0	167	40.6	365	88.8		399	97	
1	82	20	18	4.4		6	1.5	
2 or more	162	39.4	28	6.8		6	1.5	
**Systemic adverse effects**					<0.001			<0.001
Fatigue	82	20	8	2		2	0.5	
Headache	84	20.5	12	2.9		4	1	
Muscle pain	113	27.5	11	2.7		2	0.5	
Fever above 38 °C	28	6.8	2	0.5		0	0	
Fever less than 38 °C	52	12.7	3	0.7		0	0	
Nausea/vomiting	13	3.2	2	0.5		0	0	
Diarrhea	13	3.2	2	0.5		0	0	
General discomfort	137	33.3	11	2.7		1	0.2	
Widespread rash	0	0	1	0.2		1	0.2	
Rash on the face	0	0	1	0.2		1	0.2	
Chest pain	16	3.9	5	1.2		2	0.5	
Irregular pulse	12	2.9	0	0		0	0	
Difficulty breathing	11	2.7	0	0		0	0	
Drowsiness	13	3.2	2	0.5		2	0.5	
Another reaction	47	11.4	21	5.1		5	1.2	

* Difference obtained from comparing day 7 to day 3. ± Difference obtained from comparing day 14 to day 3.

**Table 3:** Bivariate analysis of the variables of interest according to the presence of short-term adverse effects in adults over 40 years of age who came to be vaccinated with the fourth dose against COVID-19 in Trujillo, Peru (N = 411).

Variables	Presence of Adverse Effects		*p*-Value
	No	Yes	
	N = 54 (13.1%)	N = 357 (86.7%)	
**Sex**			**0.043**
Female	20 (9.8)	185 (91.2)	
Male	34 (16.5)	172 (83.5)	
**Age**			**<0.001**
<60	20 (8.1)	227 (91.9)	
≥60	34 (20.7)	130 (79.3)	
**BMI**			0.858
Normal weight	12 (12.1)	87 (87.9)	
Overweight	25 (12.8)	170 (87.2)	
Obese	17 (14.5)	100 (85.5)	
**Comorbidities**			0.368
No	47 (13.8)	293 (86.2)	
Yes	7 (9.9)	64 (90.1)	
**History of COVID-19 infection**			0.229
No	41 (14.5)	242 (85.5)	
Yes	13 (10.1)	115 (89.9)	
**vaccination scheme**			**0.026**
Homologous	44 (15.7)	237 (84.3)	
Heterologous	10 (7.7)	120 (92.3)	
**Last vaccine**			
mRNA-1273 (Moderna)	45 (11.9)	334 (88.1)	**0.009**
BNT162b2 (Pfizer-BioNtech)	9 (28.1)	23 (71.9)	
**Simultaneous vaccination against influenza**			0.112
No	49 (14.3)	293 (85.7)	
Yes	5 (7.2)	64 (92.8)	
**Previous BBIBP-CorV**			**0.048**
No	46 (15.1)	259 (84.9)	
Yes	8 (7.6)	98 (92.4)	

BMI: Body Mass Index. *p*-Value obtained with chi-square test.

**Table 4:** Crude and adjusted regression models to estimate the association between the presence of adverse effects from vaccination with the fourth dose against COVID-19 and the variables of interest in adults over 40 years of age in Trujillo, Peru.

	3d vs. No Event						7d vs. No Event						14d vs. No Event					
	RRc	95% CI	*p*-Value	RRa	95% CI	*p*-Value	RRc	95% CI	*p*-Value	RRa	95% CI	*p*-Value	RRc	95% CI	*p*-Value	RRa	95% CI	*p*-Value
**Age (years)**																		
<60	Ref.			Ref.			Ref.			Ref.			Ref.			Ref.		
≥60	0.32	0.18–0.59	**<0.001**	0.29	0.15–0.53	**<0.001**	0.37	0.17–0.80	**0.011**	0.43	0.19–0.98	0.044	0.43	0.16–1.12	0.084	0.54	0.17–1.65	0.279
**Sex**																		
Female	Ref.			Ref.			Ref.			Ref.			Ref.			Ref.		
Male	0.54	0.30–0.98	**0.044**	0.53	0.28–0.99	**0.049**	0.65	0.30–1.39	0.272	0.76	0.34–1.71	0.513	0.43	0.17–1.12	0.085	0.44	0.16–1.18	0.103
**vaccination scheme**																		
Homologous	Ref.			Ref.			Ref.			Ref.			Ref.			Ref.		
Heterologous	1.88	0.90–3.91	0.092	1.31	0.59–2.90	0.510	3.96	1.67–9.37	**0.002**	3.50	1.34–9.13	**0.011**	3.23	1.14–9.11	**0.027**	2.66	0.80–8.85	0.11
**BMI**																		
Normal weight	Ref.			Ref.			Ref.			Ref.			Ref.			Ref.		
Overweight	2.34	1.12–4.89	**0.024**	0.93	0.43–2.04	0.856	2.17	0.87–5.35	0.094	1.20	0.44–3.21	0.729	4.02	1.28–12.65	**0.017**	1.47	0.43–5.01	0.524
Obesity	1.16	0.58–2.32	0.674	0.77	0.33–1.79	0.546	0.96	0.38–2.42	0.932	0.81	0.27–2.41	0.700	1.39	0.40–4.89	0.605	1.31	0.33–5.23	0.708
**Previous infection by COVID-19**																		
No	Ref.			Ref.			Ref.			Ref.			Ref.			Ref.		
Yes	1.54	0.79–3.03	0.207	1.26	0.63–2.52	0.516	1.58	0.69–3.63	0.284	1.23	0.52–2.90	0.633	0.95	0.31–2.86	0.922	0.79	0.25–2.51	0.690
**Simultaneous vaccination against influenza**																		
No	Ref.			Ref.			Ref.			Ref.			Ref.			Ref.		
Yes	2.35	0.89–6.19	0.084	2.88	1.07–7.74	**0.036**	1.84	0.57–5.88	0.306	2.27	0.69–7.52	0.178	0.82	0.15–4.52	0.817	0.85	0.14–5.03	0.856
**Presence of comorbidities**																		
No	Ref.			Ref.			Ref.			Ref.			Ref.			Ref.		
Yes	1.35	0.58–3.18	0.487	1.63	0.69–3.84	0.264	2.19	0.80–5.93	0.124	3.12	1.11–8.76	**0.031**	1.22	0.33–4.61	0.769	1.63	0.40–6.69	0.501
**Last vaccine**																		
mRNA-1273 (Moderna)	Ref.						Ref.						Ref.					
BNT162b2 (Pfizer-BioNtech)	0.42	0.18–0.96	**0.041**				0.18	0.04–0.89	**0.035**				N.A.					
Previous BBIBP-CorV																		
No	Ref.						Ref.						Ref.					
Yes	2.01	0.94–4.64	**0.071**				2.88	1.13–7.31	**0.026**				1.73	0.53–5.63	0.366			

RRc: crude relative risk; RRa: adjusted relative risk; CI: confidence interval; Ref: reference. N.A.: not available.

## Data Availability

The dataset generated and analyzed during the current study is not publicly available because the ethics committee has not provided permission/authorization to publicly share the data, but it is available from the corresponding author upon reasonable request.

## Supplementary Materials

The following supporting information can be downloaded at: https://www.mdpi.com/article/10.3390/vaccines12040400/s1, Table S1: Hazard ratio between the presence of adverse effects from vaccination with the fourth dose against COVID-19 and the variables of interest in adults over 40 years of age in Trujillo, Peru.

## Appendix A. Survey Administered on the Third Day after Vaccination with the Fourth Dose against COVID-19

**Question Numer**	**Questions**	**Conditions↑**
P1	Did you experience any side effects from the fourth dose of the COVID-19 vaccine? (Yes/No)	
P2	Did you have any local reaction in the area of your arm where you received the fourth dose of the COVID-19 vaccine? (Yes/No)	P2 = “yes”
P3	Which of the following local reactions did you have in the arm where you received the fourth dose of the COVID-19 vaccine? (multiple choice) - Pain in the injection area. - Swelling in the injection area. - Swelling in the armpit, close to the injection area. - Another reaction (Free Written Response)	P2, P3 = “yes”
P4	Did you have any local reactions in the rest of your body after the fourth dose of the COVID-19 vaccine? (Yes/No)	P2 = “yes”
P5	Which of the following adverse events did you experience in the rest of your body after being vaccinated with the fourth dose of the COVID-19 vaccine? (multiple choice) - Fatigue - Headache - Muscle pain - Fever higher than 38 °C - Fever less than 38 °C - Nausea or vomiting - Diarrhea - General discomfort - Widespread rash - Rash on the face - Chest pain - Irregular pulse - Difficulty breathing - Other reaction (Free Written Response)	P2, P3 = “yes”
Q6	How many days after the administration of the fourth dose of the COVID-19 vaccine did the adverse reactions persist? - One day - Two days - Three days	P2 = “yes”
Q7	Did you seek medical attention for one or more of the adverse effects? (Yes/No)	P2 = “yes”
Q8	Several months ago, you received the third dose of the COVID-19 vaccine, did you experience any adverse effects after the administration of the third dose of this vaccine? - Yes - No - I don’t remember	
Q9	How would you summarize your feelings in the days after receiving the fourth dose of the vaccine, compared to the days after receiving the third dose of the COVID-19 vaccine? - I feel better now - I feel similar - I feel worse now - I don’t remember	
Q10	Did you get your flu vaccine on the same day you were inoculated with the fourth dose against COVID-19? (Yes/No)	
↑ To answer the question you must have answered affirmatively the “condition questions”.		

## Appendix B. Survey Administered on the Seventh, Fourteenth, and Twenty-First Day after Vaccination with the Fourth Dose against COVID-19

**Question Number**	**Questions**	**Conditions↑**
Q1	Do local reactions persist in the area of your arm where you received the fourth dose of the COVID-19 vaccine? (Yes/No)	
Q2	Which of the following local reactions persist in the arm where you received the fourth dose of the COVID-19 vaccine? (multiple choice) - Pain in the injection area. - Swelling in the injection area. - Swelling in the armpit, close to the injection area. Another reaction (Free Written Response)	P1 = “yes”
Q3	Do local reactions persist in the rest of your body after the fourth dose of the COVID-19 vaccine? (Yes/No)	
Q4	Which of the following adverse events persist in the rest of your body after being vaccinated with the fourth dose of the COVID-19 vaccine? (multiple choice) - Fatigue - Headache - Muscle pain - Fever higher than 38 °C - Fever less than 38 °C - Nausea or vomiting - Diarrhea - General discomfort - Widespread rash - Rash on the face - Chest pain - Irregular pulse - Difficulty breathing Other reaction (Free Written Response)	P3 = “yes”
Q5	Did you seek medical attention for one or more of the adverse effects? (Yes/No)	P1 or P3 = “yes”
↑ To answer the question you must have answered affirmatively the “condition questions”.		
