# Supplementary material for: Short-Term Adverse Effects of the Fourth Dose of Vaccination against COVID-19 in Adults over 40 Years of Age
Source: Vaccines (Basel). 2024 Apr 10;12(4):400. doi: 10.3390/vaccines12040400 (PMC11055111; doi:10.3390/vaccines12040400)
Supplement: Supplementary file 1 [file vaccines-12-00400-s001.zip › vaccines-2783329-supplementary.pdf]

Table 1 Supplementary: Hazard ratio between the presence of adverse effects from vaccination with the fourth dose against COVID-19 and the variables of interest in adults over 40 years of age in Trujillo, Peru.

	HR	95% CI	p-value
**Age (years)**			
<60	Ref.		
≥60	0.77	0.67 – 0.89	**<0.001**
**Sex**			
Female	Ref.		
Male	0.88	0.77 – 1.00	**0.060**
**vaccination scheme**			
Counterpart	Ref.		
heterologous	1.09	0.96 – 1.23	0.198
**BMI**			
normal weight	Ref.		
Overweight	0.96	0.82 – 1.13	0.636
Obesity	0.94	0.78 – 1.13	0.533
**Previous infection by COVID-19**			
No	Ref.		
Yes	1.11	0.97 – 1.27	0.144
**Simultaneous vaccination against influenza**			
No	Ref.		
Yes	1.20	1.04 – 1.40	**0.016**
**Presence of comorbidities**			
No	Ref.		
Yes	1.06	0.90 – 1.23	0.516
**Last vaccine**			
mRNA-1273 (Moderna)	Ref.		
BNT162b2 (Pfizer-BioNtech)	0.78	0.55 – 1.11	0.169
**Previous BBIBP-CorV**			
No	Ref.		
Yes	1.14	1.01 – 1.30	**0.049**

HR: Hazard Ratio. CI: confidence interval; Ref: Reference
